# Lecture on Clinical Medicine, Delivered at the Philadelphia Medical Institute

**Published:** 1838-10-24

**Authors:** W. W. Gerhard

**Affiliations:** Physician to the Philadelphia Hospital, &c.


### CLINICAL LECTURE.

LECTURE ON CLINICAL MEDICINE, *delivered at the Philadelphia Medical Institute, by* W. W. GERHARD, M. D., *Physician to the Philadelphia Hospital*, &c.

### ORGANIC DISEASES OF THE HEART.

IN my last lecture, I concluded the subject of the inflammations of the membranes of the heart; we now naturally come to treat of the organic affections of this organ. There are three great causes of organic disease of the heart: 1st, inflammation of its membranes, especially the internal coat; 2d, functional disorder of the heart, if long protracted; 3d, the physiological changes occurring during the progress of life. Now, you may regard all cases of organic disease of the heart as produced by one of these series of causes, or by the concurrent action of more than one of them.

The most frequent cause of heart disease is probably inflammation, particularly in the earlier years of life. At one period I was disposed to underrate the influence of inflammation; but upon a closer and more continuous examination of the subject, I have been convinced that this cause is not only one of the most frequent, but also that it is more powerful and more immediate in its operation than either of the others. You must have seen enough of the progress of this form of disease, during the present course, to convince you of its frequency; and if you followed out the history of the cases with me, you must have seen that we arrived at our conclusions by the double chain of reasoning which affords the only approach to certainty in medicine. That is, we examined many cases of diseased heart very early during the active inflammatory period, and observed them to glide insensibly into organic lesions; while we met with other cases which had passed through the earlier stages of the disease before coming under our notice. In the latter class of cases, by careful investigation, we were enabled to trace back the succession of symptoms, until we arrived at an acute attack, previously to which no inconvenience whatever had been sustained by the patient. With the precautions we took, there was little probability of error; at any rate, there could be no serious error, for the patients, if not entirely well, were so nearly in a state of health, as to be unconscious of any unusual symptoms. The disease, therefore, was at least awakened from a latent into a decided form, by the action of an inflammatory cause. I believe, however, that in the large majority of these cases, there was no previous disease,—but that after the affection of the heart had once originated, its progress became in some degree independent of the original exciting cause, and, like all diseases of this organ, it is the cause of its own increase. Articular rheumatism very often gives rise to acute inflammation of the heart, and in this way becomes the starting point from which we may trace its organic lesions.

There is, however, another mode in which the irritation of rheumatism acts directly upon the heart without giving rise to positive inflammation. Thus, if a patient has been subject to transient rheumatic pains, which are renewed very frequently upon change of weather, and which shift suddenly from one portion of the fibrous tissue to another, you may from time to time hear him complain of acute pain in the heart, often shooting through this organ, and frequently accompanied with palpitations; now, as the palpitations and the pain become frequent, they give rise to a real increase in the size of the heart, and which may be in time followed by alteration of its valves. Thus, you may remark, that a slight degree of irritation produced by a rheumatic diathesis, is productive of disease of the heart, which is, however, of less immediate importance than that which follows an attack of acute inflammation. The valves may often escape until a late period, and the whole disease is slower in its progress.

The second cause of organic disease of the heart is functional disorder of this organ; that is, when long protracted. You have seen comparatively few cases of this kind, although they are by no means rare. The functional causes are very numerous; intermittent fever, a disorder of the spinal nerves, chronic uterine disease, anemia, violent exercise, in short any disease which keeps the heart in a state of violent and very frequent action must in the end give rise to a disorder of this organ, which is usually dilatation. These cases are scarcely ever so severe as to require admission into an hospital, except on account of some other disease under which the patient is labouring; hence you scarcely ever see them in the uncomplicated form best adapted for study.

We see them at the close of intermittents which have broken down the constitution of the patient, or we observe them in the course of another disease which is somewhat aggravated by the alteration of the heart, particularly if the lungs should be the seat of it. The close connexion existing between these organs sufficiently explains the increased suffering which is necessarily produced by the double alteration. In hospitals, these cases are frequently seen as complications of disease of the lungs, particularly chronic bronchitis and emphysema. Disease of the heart is so frequent in these cases that you may often hear some confidently affirm the existence of dilatation, either simple or combined with hypertrophy, in patients who become asthmatic after a long continuance of chronic dry catarrh. In phthisis, the complication is by no means unfrequent, but it is confined to dilatation most frequently, and the tuberculous affection is much more developed in the left side than in the right; the patient suffers extremely from the violence of the palpitation. The condensed tissue of the left lung is, in these cases, placed immediately in contact with the heart and serves as a conductor of the impulse, while, at the same time, it materially increases its force by furnishing a solid tissue for the heart to impinge upon, instead of a soft elastic lung.

I have already stated that this form of disease is usually free from valvular lesion, and that it therefore interferes but moderately with the action of the heart. It may often be removed, in a great degree, by art, and when not susceptible of entire cure, may be so much relieved as to leave the patient in a state of comparative health. When you reflect upon the tendency of functional disease to terminate in organic lesion, the question may here naturally occur to you, whether it is possible to distinguish the moment when the disorder of the heart ceases to be merely functional. There is some difficulty in this matter, but, as you will afterwards learn, you may, with careful examination, separate the two forms of disease in almost every instance.

The last cause of disease of the heart, is that produced by the gradual physiological changes which occur in advanced life. This subject has been lately set in a very clear light by my friend Dr. Bizot, of Geneva, (see *Memoires de la Societé Médicale d'Observation.*) He has shown very satisfactorily that there is a necessary increase in the thickness of the parietes of the heart, after the middle age of life. He has, besides, demonstrated the gradual progress of cartilaginous and bony alteration of the valves, and of the larger arteries in aged patients. In certain individuals, particularly those of a gouty diathesis, the ossific deposit occurs to a much greater degree, and at an earlier period, than in the rest of mankind; hence, such persons are especially liable to disease of the heart with alteration of the valves. This lesion, in those individuals, is quite independent of inflammation, and is remarkable for its frequent occurrence in certain families, every member of which is sometimes cut off by sudden death from heart disease, after reaching a particular age. Of course, this variety is quite incurable, and all that we can do is to direct such hygienic means as may diminish the chances of a speedily fatal termination, and to recollect the complication of heart disease when these patients are attacked with an acute disease of a different kind, for our treatment may then require some modification.

You have seen the influence of all these causes of disease of the heart, and to you, my lecture is little else than a clinical demonstration; although, from the nature of the subject, I am compelled to throw a part into a form somewhat different from an ordinary clinical lecture. The subject is too complex for a clear understanding of it without some accurate previous knowledge, but if you examine your observations, after a full acquaintance with the physiology and pathology of the heart, you will find its disease characterized by great simplicity, and that, with slight exceptions, they are very readily recognised. Now, I do not intend to go very minutely into this matter, but I wish to render the subject clear enough for you to understand it.

We next come to the examination of the morbid conditions of the heart, which are regarded as organic diseases. These are thickening of the valves from a morbid deposit of cartilage or bone in their substances, and vegetation or thickening of their lining membrane, agglutination of the surfaces of the valves, and ulceration. The deposition of cartilage or bone takes place in aged persons, from an altered physiological condition, but under other circumstances you may regard all those lesions of the valves as the result of endocarditis. The exceptions to this rule are extremely rare.

Most severe organic diseases of the muscular substance of the heart commence by an alteration of the valves. This depends upon the fact, that any alteration in the outlet of a hollow viscus, necessarily brings about a change in the structure of the organ itself. Now you may regard the law as settled, that, "if a patient be robust and his nutrition be tolerably active," the viscus is dilated, and, at the same time, thickened. The increase of thickness of its walls merely keeps up the proportion between the size of the whole organ and its strength. If, however, the nutrition of the patient be enfeebled, you will find that this is not the case; the organ dilates, but there is no compensating thickening, hence the cavity of the organ is increased, while its power of contraction is decidedly diminished. Now, an obstacle to the passage of the blood from thickening or morbid growth upon a valve, may be regarded as a necessary cause of dilatation of the heart, which, in most subjects, is, at the same time, combined with hypertrophy. I need not now call your attention to several patients in whom this process very obviously occurred, amongst them is Robb, to whose case I have so often alluded. This man, after his recovery from acute inflammation, in addition to the valvular disease, was, undoubtedly, attacked with hypertrophy and dilatation.

I just spoke of contraction of the valvular opening, as a cause of dilatation and hypertrophy of the heart, from the distention of the heart with blood, which passes slowly through a narrow orifice, and from the constant motion of the heart to get rid of the distending mass. Like all the muscular tissues, it is thickened from excess of action, but dilated from a cause which is almost purely mechanical. The morbid dilatation of the valves, or patescence, as it is sometimes called, is, however, attended with nearly the same results as their contraction; although the mechanism of the symptoms is somewhat different in the two cases.

When a valve is permanently contracted, the cavity of the heart containing the blood which is to pass through it necessarily labours with increased energy in an effort to force a given quantity of blood through a contracted opening, in the same time which was previously occupied by its passage through a larger orifice. The power of the muscular contraction must of course be proportioned to the size of the opening, the quantity of blood, and the vigour of the patient; if an accidental functional disorder of the heart should take place, this organ is thrown into a violent and temporary contraction, and the circumstances generally controlling its action are of course somewhat modified, so that the heart of a feeble patient, who happens to be much excited, may for the time beat with greater violence than that of an individual possessing much greater muscular strength. In a disease of the valves which prevents their closure, the rationale of the process by which the heart itself becomes dilated and hypertrophied, is not very different from that by which the same change follows contraction of the valves. The valves do not close completely; a portion of the blood which has just been expelled from a cavity, returns to it and dilates it beyond its usual limits; the heart must again contract with increased energy, for it has to propel not only the requisite quantity of blood, but another portion which does not pass forward in the general current that returns back to the same point from which it had started. Hence, on this account, whether the valves be dilated or contracted, a very similar change occurs in the conformation of the cavity which discharges itself through them, and the general symptoms of the two kinds of valvular alteration differ but little from each other, except in severity. Those arising from dilatation of the valves are, as a general rule, less intense than those which depend upon contraction, but in practice these two lesions are usually formed together, and the symptoms are somewhat complicated. The coincidence of the two lesions arises from the circumstance that a thickened valve projects towards the interior of the cavity of the heart, and from its rigid, unyielding texture neither dilates fully when the blood is to pass through the orifice, nor is it as capable of completely closing it when the current of blood should be interrupted.

The symptoms of valvular disease I have already demonstrated to you when lecturing upon endocarditis. The signs which depend upon permanent disease of the valves, differ but little from those caused by the temporary thickening arising from a deposit of lymph between the two layers of lining membrane, or upon its free surface. You will find in both cases, the same roughness of the first sound of the heart, which amounts to a decided rasping sound, if the deposit consist of projecting excrescences or of bony spicula, while there is only a bellows sound, if the valves are simply cartilaginous and still flexible. The second sound is enfeebled, or entirely lost. These signs of course apply to contraction of the orifices. The roughness accords with the first sound of the heart, because the contracted valve presents an obstacle to the passage of the blood during the systole, which as you know gives rise to the first sound. The second sound, is entirely caused by the sharp contraction of the valves; hence, any cause retarding or preventing that contraction, tends to enfeeble this sound.

We have at this moment, several cases of the kind, and almost always can present you with illustrations, chiefly amongst the patients most advanced in age. I would refer you particularly to Elfrey, as a very appropriate example of intense rasping sound in the systole, and very faint sound during the diastole of the heart.

You will almost always find that the rasping sound, occurring during the systole, arises from vegetations or bony deposits at the semi-lunar valves of the aorta; they must impede the passage of the blood from the left ventricle into the aorta, and thus give rise to the rasping sound. But the semi-lunar are not the only valves whch may produce a rasping in the first sound. If the mitral valve remains widely open during the systole, the column of blood may be forced backwards from the ventricle. It will then produce very nearly the same sound as if it were repulsed by some obstacle to its passage arising from the projection inwards of the aortal valve. The alteration of the sound caused by the regurgitation of the blood from the ventricle, is however much less striking than that arising from contraction of the semi-lunar valves, and therefore less easily recognised.

There is another form of simple valvular disease which you should recollect; that is, the patescence or dilatation of the aortal valves, without any obvious contraction. The effort of this is, of course, to allow the blood to pass both through the mitral and aortal valves during the dilatation of the ventricle. Neither sound of the heart is lost, but they are somewhat confused; the second sound looses its clearness, and very nearly resembles the first. The dilatation of the valves to which I allude, occurs chiefly in hypertrophy and dilatation of the heart, and is rather a secondary than a primary lesion.

Vegetations upon the mitral valve sometimes occur, and interfere more or less with its functions, without necessarily impeding the passage of the blood through it. I once saw a case of this kind, in which there was no marked alteration of either sound, but merely extreme irregularity of the heart's action, recurring in paroxysms. These would again cease, and the patient be left in quiet for several hours together. Agitation of any kind would remove these palpitations. There was a concretion attached to the side of the mitral valve, of the size of a small hazle-nut; not sufficiently to prevent the passage of the blood, but large enough, at times, to impede the action of the valve. Probably, when the heart was beating in a very regular manner, the lesion was of little consequence, but when the activity of the circulation was such that the valve must close with great rapidity, an impediment of the kind formed a serious obstacle to the passage of the blood. The patient was attended by my friend, Dr. Hays, at whose request I made the examination of the body.

Contraction of the mitral valve, is a very serious lesion of the heart, and when it extends to a great degree, must cause a roughness during the diastole of the heart. But if the aortal valves remain healthy, you will be not a little puzzled; for you may detect a normal second sound, more or less mingled with the roughness caused by the passage of the blood into the ventricle during the diastole of the heart. You will find a difficulty in understanding this subject, and I think it better to postpone the consideration of it to another lecture, as I think it essential for you to study the diseases of the heart in their simplest forms, before proceeding to the cases which are more obscure.

